# Never Say No … How the Brain Interprets the Pregnant Pause in Conversation

**DOI:** 10.1371/journal.pone.0145474

**Published:** 2015-12-23

**Authors:** Sara Bögels, Kobin H. Kendrick, Stephen C. Levinson

**Affiliations:** 1 Language and Cognition Department, Max Planck Institute for Psycholinguistics, Nijmegen, The Netherlands; 2 Donders Institute for Brain, Cognition and Behaviour, Nijmegen, The Netherlands; Max Planck Institute for Human Cognitive and Brain Sciences, GERMANY

## Abstract

In conversation, negative responses to invitations, requests, offers, and the like are more likely to occur with a delay–conversation analysts talk of them as dispreferred. Here we examine the contrastive cognitive load ‘yes’ and ‘no’ responses make, either when relatively fast (300 ms after question offset) or delayed (1000 ms). Participants heard short dialogues contrasting in speed and valence of response while having their EEG recorded. We found that a fast ‘no’ evokes an N400-effect relative to a fast ‘yes’; however, this contrast disappeared in the delayed responses. 'No' responses, however, elicited a late frontal positivity both if they were fast and if they were delayed. We interpret these results as follows: a fast ‘no’ evoked an N400 because an immediate response is expected to be positive–this effect disappears as the response time lengthens because now in ordinary conversation the probability of a ‘no’ has increased. However, regardless of the latency of response, a ‘no’ response is associated with a late positivity, since a negative response is always dispreferred. Together these results show that negative responses to social actions exact a higher cognitive load, but especially when least expected, in immediate response.

## Introduction

In the fall of 2013, in the Toronto city council chamber the following exchange took place:

Council Member: *Have you purchased illegal drugs in the last two years?*

(8.0 second silence)

Mayor Rob Ford: *Yes I have*.

The long pause was as much a major focus of the ensuing news reports as the revelation (e.g., [1, 2]). In this paper we explore experimentally with EEG (electro-encephalography) the semiotics of the pregnant pause and the expectancies that lie behind it.

Most natural language use occurs in interactional or conversational contexts, in which speakers take short turns at talking. This rapid response system relies on at least two organizational structures: the turn-taking system [3], on the one hand, and the tying of paired turns as in question-answer, request-compliance, greeting-greeting, and so forth, on the other [4]. The turn-taking system has come under increased scrutiny recently–we now know that the cross-linguistic average response time is about 200–300 ms, much faster than language production latencies, implying early prediction and response (see [5] for review).

The other crucial system, the system of paired turns, organizes an initiating action (speech act) and its possible responses–for example, an acceptance or rejection following an offer. It turns out that the responses after such an initiating action are rarely equal: the initiating action is constructed to expect a particular response, such as an acceptance of an offer or compliance with a request. In the unmarked case, the expected response is positive, such as an acceptance or compliance, and it tends to have a simple form, like *yeah*, *sure*, or *thanks* [6]. In contrast, negative responses which reject or decline the initiating action tend to be delayed in time, may occur with turn-initial particles like *uh* or *well*, and include accounts for the negative response (e.g., *uh no*, *I’m a bit tired actually*) [6]. Indeed, the general reluctance to give negative responses is highlighted in criticisms of the “no means no” campaign against sexual assault [7] and the recent shift to “yes means yes” [8]. This structural and temporal asymmetry, and its association with the response that the initiating action is built to expect, has been called *preference organization* [9] (p. 332–9). Positive responses in line with the expectation displayed in the initiating action are called *preferred* responses and negative responses that block the initiating action are *dispreferred* responses. *Preference* in this sense is not to be confused with psychological desire–I may feel obliged to offer you the last of my chocolates even if I hope you don’t take it. Importantly, in conversational corpora, preferred responses are twice as frequent as dispreferred ones ([10] give the proportion as 63% vs. 37%).

In this paper we are particularly interested in one mode of marking preference–immediacy or delay in response. It was first noted in qualitative research [11, 12] that preferred responses normally occur quickly after the initiating action, whereas dispreferred responses are more often delayed. Such a delay can be instantiated by turn-initial particles and hesitations and/or by a longer gap before the response. It appears that such delays can also shape expectations about the upcoming response, as illustrated by the following example.

JS:II:48 [11] (77)

A: *D’they have a good cook there?*

(1.7 second silence)

A: *Nothing special?*

B: *No*. *Every- everybody takes their turns*.

Here, a delay of 1.7 seconds after a question leads the questioner to revise her question such that a negative response becomes preferred. This suggests that the questioner inferred (correctly) from the delay that the response to her first version would probably be dispreferred. Thus, as Clayman [13] notes, “interactants can anticipate the type of response that is forthcoming purely on the basis of its initial form. . . . any delay in responding–even mere silence–may be interpreted as the first move toward some form of disagreement/rejection” (p. 235).

Supporting these qualitative observations, in a large quantitative study on the timing of turn-taking in 10 different languages [14], confirmations to questions were found to occur faster on average than disconfirmations (significantly in 7 out of the 10 languages). And answers were given faster than non-answers (in all 10 languages), the latter arguably being a type of dispreferred response [15]. Kendrick and Torreira [10] examined the complete distribution of response timings in another quantitative study on English, regarding requests, offers, invitations, proposals, and suggestions only. They found that preferred responses mostly occurred after short gaps, but as the delay mounted to around 700 ms, dispreferred responses became more frequent than preferred ones.

The judgments that listeners make of responses based on their timing have also been investigated experimentally [16–18]. Participants heard constructed telephone conversations ending in a request or assessment followed by differing amounts of silence and then a positive response (*yeah* or *sure*). Participants were asked to judge how willing the respondent was to agree with the request or assessment. Responses after longer silences were judged as less willing than responses after shorter silences. In summary, earlier studies suggest that after a long gap, a dispreferred response may be more expectable than a preferred response. However, it remains unclear whether listeners use this information on-line to form cognitive expectations about the upcoming response. The present study explores this, using electro-encephalography (EEG), which has the special advantage of allowing millisecond-by-millisecond monitoring of mental activity without requiring a behavioral response.

We note here that response latency might also have other functions than indicating preference. Longer latencies have been related to lower speaker's confidence (or 'feeling of knowing') in answering a factual question [19] and listeners judge this in a similar way [20]. Speakers also produce longer silences before lies than before telling the truth [21, 22], which is also picked up by listeners if they have to distinguish between the two [22]. However, this literature refers to responses to questions asking about facts and personal events, which are of a different nature than responses to requests, invitations, and the like, which solicit commitments to future courses of action.

### Previous EEG research on the comprehension of interactive language use

In work on language comprehension, one important method uses event-related brain potentials (ERPs), EEG responses to specific events. ERPs provide a sampling of the brain’s electrical activity with a high temporal resolution. ERP studies on language comprehension have made extensive use of the N400 component, which has been associated with semantic processing. This is a negative going wave in the ERP, elicited by all words but to differing amounts, that peaks around 400 ms after word onset. The amplitude of the N400 has been found to be smaller when it fits the sentence context better (e.g., [23]). The exact interpretation of the N400 is still under debate, since it could reflect the amount of active prediction of words on the basis of the context (e.g., [24]) and/or the ease of integration of a word in the context once it is encountered (e.g., [25]). Following Van Petten and Luka [26], we use the term *expectation* to refer to anticipation of general semantic content on the basis of the context, which is then integrated with the word upon encountering it.

The N400 has been shown to be sensitive not only to the semantic content of linguistic expressions but also to their match to expectations in the real world (e.g., [27]). Going beyond the single sentence, Van Berkum, Hagoort, and Brown [28] found that the N400 amplitude was also sensitive to the degree of fit with a larger story context. Although much of this research has been done with written stimuli, similar results have been obtained with spoken language (e.g., [29]). In addition to the N400 component, ERP studies on language comprehension have also made use of late positivities, which have been related to reanalysis or further processing in language comprehension. Late positivities have been found in response to pragmatic phenomena [30–33] and social norm violations [34, 35], among others.

Research on dialogue using EEG is in its infancy. In addition to ERPs, researchers have also begun to examine the time frequency of oscillatory activity in the brain to investigate dialogue. For example, work on expectations about when a turn at talk will end found desynchronization of beta oscillations in the context of more predictable turn-endings [36]. A study investigating when during a turn participants start to prepare their responses showed a positive component in the EEG soon after the moment they could start retrieving the answer, which was localized to language production areas, as well as desynchronization in the alpha/beta range [37]. Participants listening to constructed dialogues showed early differential ERP effects for responses performing different speech acts [38], indicating that speech acts can be recognized early. In this research an attempt is made to use materials as close to actual conversational models as possible. Magyari and colleagues [36] used turns extracted from corpus recordings, while Bögels and colleagues [37] used an interactive quiz paradigm with partially pre-recorded questions to engender what for the recipient appeared to be real dialogue. In the present study we extracted initiating actions from a corpus of telephone calls, cross-splicing responses from elsewhere in the corpus.

### The present study

We set out to examine whether the expectation of a specific type of response is affected by (1) the preference structure set by the initiating action and (2) the timing of the response. To investigate this, we extracted from a corpus of Dutch telephone calls initiating actions (requests, offers, proposals, and invitations; see Materials), which make relevant acceptance (granting, agreement, etc.) as the preferred response, as based on the conversation analytic literature (e.g., [12]). These initiating actions were followed by either a short gap of 300 ms or a long gap of 1000 ms. These values were chosen because 300 ms approximates an average gap duration [39] and 1000 ms is a long gap after which dispreferred responses are more frequent than preferreds (for example, in a study on English [10], approximately two-thirds of responses delayed by 700 ms or more were found to be dispreferred). The gap was followed by a response extracted from elsewhere in the corpus. The responses were short and restricted to either *ja* ('yes') for a preferred response or *nee* ('no') for a dispreferred response in order to control for the complexity of the response and to compare ERPs across conditions. Although dispreferred responses often include additional elements (e.g., turn-initial particles like *well* in English or *nou* in Dutch, and hesitations such as *uh*), simple 'no' responses do occur in conversation, taking up 25% of all dispreferred responses in one corpus [10]. Still, the absence of additional elements, which was necessary for experimental control, might add to the unexpectedness of the dispreferred responses in the present study.

We hypothesized (H1) that listeners should take into account the preference structure set by the initiating action and therefore that a preferred response ('yes') should be more expected than a dispreferred response ('no'). This expectation should hold specifically after a short gap (300 ms). In terms of ERP effects, we hypothesized an N400 effect for dispreferred relative to preferred responses after a short gap. In addition, we hypothesized (H2) that since dispreferred responses are more frequent than preferreds after long gaps [10], a dispreferred response (‘no’) should be less exceptional after a long gap (1000 ms) than after a short one. Thus, we do not expect any N400 effect for 'no' after a long gap. An even stronger version of this hypothesis (H3) is that of a cross-over interaction: after a long gap a 'yes' might be less expected than a 'no', leading to an N400 effect for preferred relative to dispreferred responses in this context. This last hypothesis is less certain since a general normative preference for preferred responses [40] might operate in parallel with the effect of the longer gap, possibly resulting in the two effects cancelling each other out. In addition to N400 effects, we also thought we might find differences between the conditions in terms of late positive effects, which have been associated with the further processing involved with less expected responses [41–44]. Dispreferred responses might elicit late positivities because of their disaffiliative nature, given that for example reading about morally unacceptable behavior has also led to such effects in an earlier study [34].

## Materials and Methods

### Participants

The experiment was approved by the *Ethics Committee Faculty Social Sciences* of the Radboud University Nijmegen. Thirty-four participants (8 males) from the database of the MPI for Psycholinguistics took part in the experiment. Two were excluded from the final analysis due to excessive artifacts or mistakes in the task (see Methods). The 32 remaining participants (8 males) were 21.8 years old on average, right handed, and native speakers of Dutch without reading or hearing problems. They were paid 8 euros per hour for their participation.

## Materials

We used requests, offers, proposals, and invitations as our initiating actions because they form a natural class [10, 12, 45]. These actions all refer to a future activity to which the recipient should either commit (preferred response) or not commit (dispreferred response). The telephone conversations of the Corpus of Spoken Dutch (CGN [46]) were examined for any requests, offers, proposals, and invitations which met the following criteria. First, the actions impressionistically sounded intonationally and pragmatically complete (see [47]). Second, the future activities involved the speaker and the recipient, not a third party. Third, only questions were included to ensure that a response was conditionally relevant. Fourth, the grammatical formats were not biased towards a negative response [48]. That is, the questions did not contain negative words (as in *kan je niet vragen*… 'can't you ask…') or added negative (tag) questions (as in *of zeg je*, *nee toch maar niet?* 'or would you say, no actually not?'). We selected 120 initiating actions that met these criteria: 14 invitations, 25 requests, 61 proposals, and 20 offers. Additionally, 30 *ja* ('yes') and 30 *nee* ('no') tokens were selected from different positions in the same corpus. They had a neutral intonation (e.g., not hesitant or questioning) and sounded intonationally complete.

For all experimental and practice items, a short written context of 1 or 2 sentences was created for participants to read before hearing the item (see Procedure and Table 1). If possible, the original context of the initiating action in the telephone conversation was described. If not (e.g., because the previous turns concerned a different topic or because the situation was highly complex), a plausible context was constructed.

**Table 1:** Example of an item in one of the four conditions (300 ms gap, yes response) in Dutch, with English translations, including the preceding written context and an example of a comprehension statement that occurred in 20% of trials (see Procedure).

Context (written)	Initiating action	Gap	Response	Statement (task)
*De spreker praat met een vriend over de nieuwe baan van de vriend*, *waardoor hij deze week heel druk is*. 'The speaker talks with a friend about the latter’s new job causing him to be very busy this week.'	*heb je volgende week nog een uh een moment om ons te ontvangen?* 'Do you uh have a moment next week to receive us?'	300 ms	*ja* ('yes')	*De spreker wil graag vandaag nog bij de vriend op bezoek*. 'The speaker would like to visit the friend today.'

The conditions were created by cross-splicing either 300 ms or 1000 ms of background noise from the same recording after each initiating action, followed by either a *ja* or a *nee* response token (see Table 2). For the response, from the stereo signal only the channel with the response was used; in the other channel the same background noise was inserted. Using background noise from each recording was necessary since the variability of noise in the recordings was high (adding to the naturalistic nature of the stimuli). The initiating action and the response were always present in different channels. *Ja* and *nee* tokens from speakers of the same gender were paired up and each pair was coupled to four initiating actions (in different conditions). As a result, each participant heard every response token only twice (see Design).

**Table 2:** Design with four conditions.

Initiating action	Gap	Response
Turn	300 ms	*ja* ('yes')
Turn	300 ms	*ja* ('yes')
Turn	1000 ms	*nee* ('no')
Turn	1000 ms	*nee* ('no')

For the practice block, ten initiating actions (suggestions and requests for information) that could receive a 'yes' or 'no' response were selected from the same corpus. Half were followed by the original *ja* or *nee* response from the corpus; and half were followed randomly by between 0 and 1000 ms of background noise and a cross-spliced response (see above).

### Design

The two factors gap duration (300 ms, 1000 ms) and response type ('yes', 'no') were fully crossed to create 4 conditions (see Table 2). Four lists were created, all administered to one fourth of the participants. Each list contained all 120 items once, in the same order, 30 in each condition. The conditions were rotated over the items in the 4 lists using a Latin Square design. The 120 items were divided into 3 blocks of 40 items, with pauses in between. Each block contained 10 items of each condition in a semi-random order, with the following restrictions. The same response token extracted from a particular recording was always separated by at least 4 items and occurred only twice in each list. Initiating actions coming from the same telephone conversation were separated by at least 3 items. The same condition appeared maximally twice in a row.

### Procedure

After having given written informed consent and receiving EEG preparation, participants sat down in a sound proof booth in front of a computer screen. They read the instructions on the screen and could ask questions afterwards. They were instructed that they would hear fragments from a corpus of telephone calls. They were told that fragments had been selected in which the speakers were making plans and in which the response was 'yes' or 'no', and that a written context would be provided before each fragment to help them understand the fragment. For each trial, participants first read the written context on the screen and pressed a button to continue. Then, a fixation cross appeared and after 1000 ms the fragment played. The fixation cross disappeared 1000 ms after the end of the fragment, followed by a written statement in 20% of the items (and 50% of the practice items). See Table 1 for an example. Participants indicated whether they thought the statement was true (left button) or false (right button). On average, only 2 (out of 25) statements were responded to incorrectly (range: 0–6), indicating that participants paid attention during the experiment. One participant who made 7 mistakes was discarded from the analyses. Finally, a blinking sign was presented on the screen for 2000 ms, asking participants to blink. They were instructed not to move, blink, or move their eyes while the fixation cross was on the screen.

The experiment started with a practice block of 10 items, after which participants could ask questions and received feedback about their blinking. The practice and the three experimental blocks together lasted about 50 minutes. At the end of the experiment, participants filled out a short questionnaire on the computer. In this questionnaire, only one participant reported to notice anything with regards to timing of responses (some came late and some early). The experiment, including EEG set-up, lasted about 2 hours in total.

### Apparatus

EEG was recorded from 61 active Ag/AgCI electrodes using an actiCap (e.g., [36]). Of these, 59 electrodes were mounted in the cap with equidistant electrode montage referenced to the left mastoid. Two separate electrodes were placed at the left and the right mastoid outside of the cap. Blinks were monitored through a separate electrode placed below the left eye and one of the 59 electrodes in the cap. Horizontal eye movements were monitored through two separate electrodes placed at each outer canthus. The ground electrode was placed on the forehead. Electrode impedance was kept below 10 kΩ. EEG and EOG recordings were amplified through BrainAmp DC amplifiers. EEG signals were filtered online with a band-pass filter between 0.016 and 100 Hz. The recording was digitized online with a sampling frequency of 500 Hz and stored for offline analysis.

### Data analysis

Preprocessing and statistical analysis of EEG data was conducted using Fieldtrip [49]. First, epochs were extracted from the EEG from 500 ms before the start of a response until 1000 ms after. For purposes of artifact rejection, these epochs were filtered with a low pass filter of 35 Hz, detrended, and baselined with a baseline of 200 ms immediately before response onset. Epochs containing eye artifacts or other artifacts that exceeded +/- 100 μV (visual inspection) were discarded. One participant with less than 24 remaining trials in some of the conditions was not analyzed further. For the remaining participants, an average of 28 or 29 trials (range: 24–30) remained for all four conditions (no significant differences between the conditions, p > .25). These clean trials entered the ERP analyses. Epochs were low-pass filtered at 35 Hz and baselined with a baseline window of 200 ms immediately before response onset. Trials of the same condition were averaged per participant.

To test for statistically significant differences between conditions, we used the cluster-based approach implemented in the Fieldtrip toolbox [50]. This robust method reduces the multiple-comparisons problem and controls family-wise error across subjects in time and space (see [37] for a full description of the procedure). We first tested for interactions between gap duration and response type, both in the complete time window between 0 and 1000 ms and in a pre-specified N400 window used in previous studies (300–500 ms, e.g., [28]). Within the cluster-based approach this was done by calculating the mean difference between the 'yes' and 'no' responses for each participant within the 300 ms and 1000 ms gap conditions. Then the cluster-based approach was used on these difference scores with the within-subject factor gap duration. In the case of an interaction, separate analyses were performed to compare 'yes' and 'no' responses within the 300 ms and 1000 ms gap conditions.

## Results

**Fig 1:**
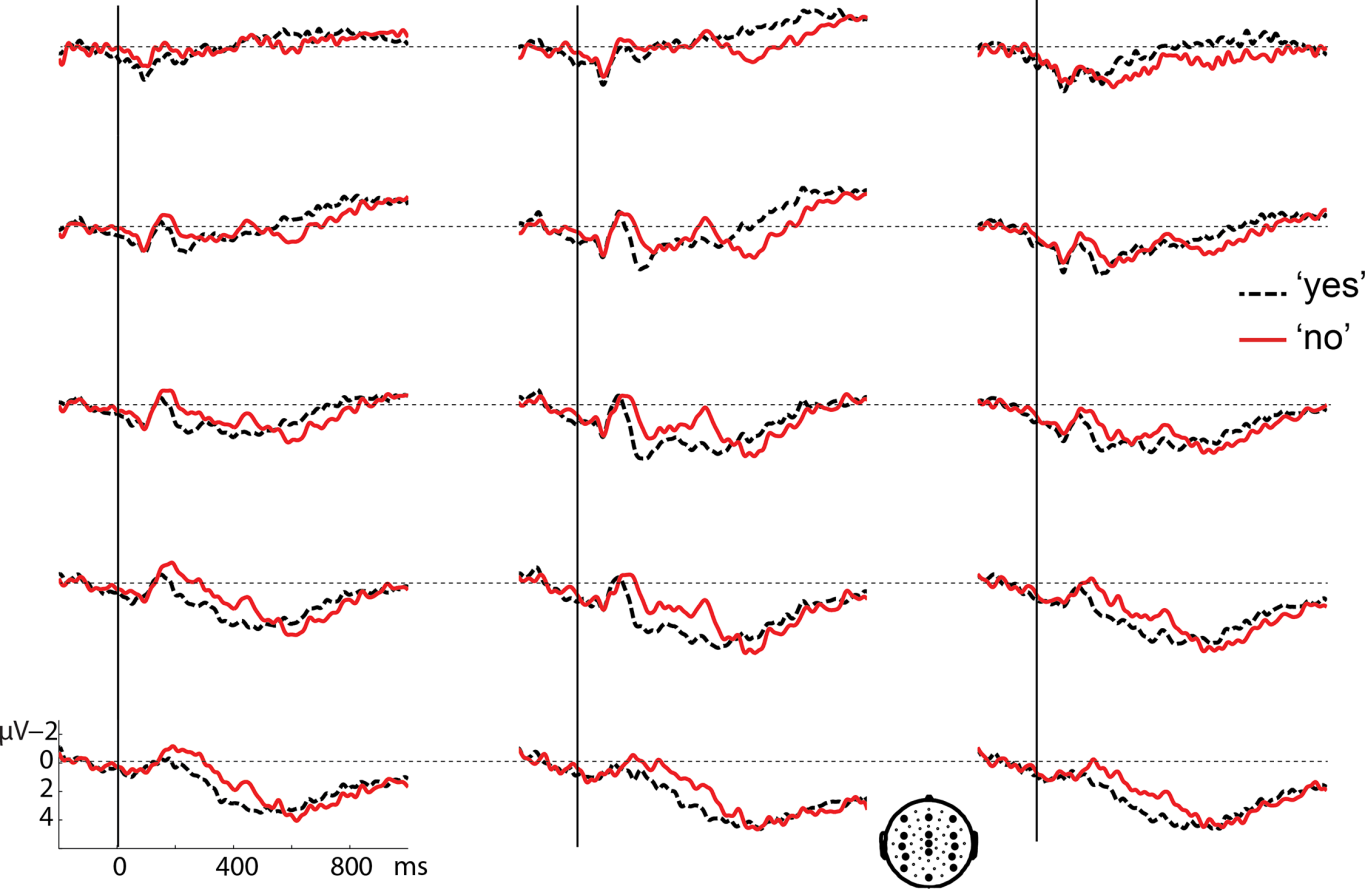
Grand average waveforms for the 'yes' response (black dashed line) and the 'no' response (red solid line) after a 300 ms gap, time-locked to response onset. A representative subset of 15 electrodes is shown, the locations of which are indicated on the head at the bottom.

**Fig 2:**
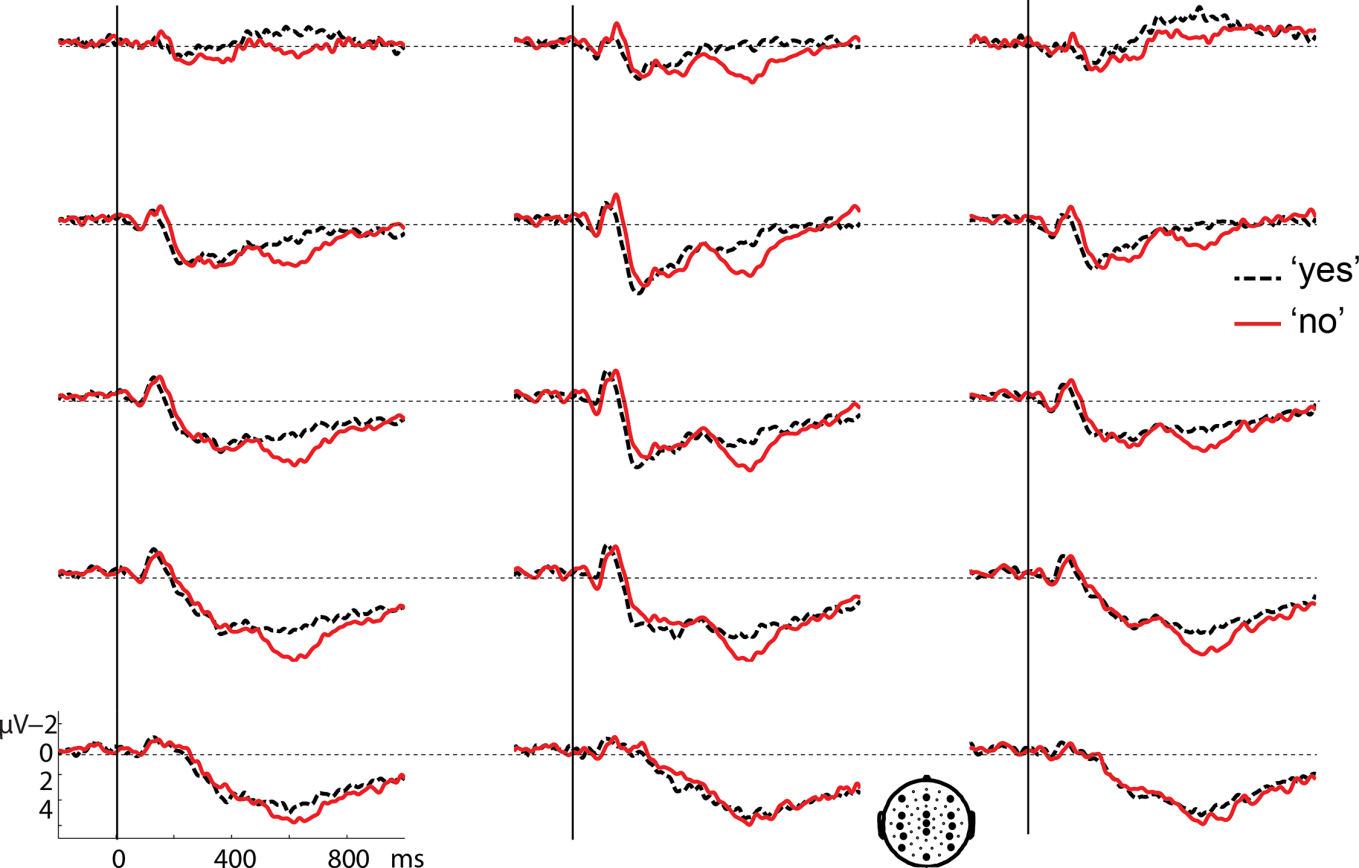
Grand average waveforms for the 'yes' response (black dashed line) and the 'no' response (red solid line) after a 1000 ms gap, time-locked to response onset. A representative subset of 15 electrodes is shown, whose locations are indicated on the head at the bottom.

Figs 1 and 2 present grand average waveforms time-locked to the onset of the 'yes' and 'no’ responses after a 300 ms gap (Fig 1) and a 1000 ms gap (Fig 2). An N400 ERP effect appears present in centroparietal electrodes for 'no' relative to 'yes' after a 300 ms gap, starting around 150 ms after response onset (see Fig 1). In contrast, no effects appear to be present around this time window after a 1000 ms gap (see Fig 2). About 500 ms after response onset, a positive effect appears to emerge for 'no' relative to 'yes' in both figs. Its distribution is predominantly anterior in both conditions, but seems larger and more widespread after the 1000 ms gap (Fig 2).

**Fig 3:**
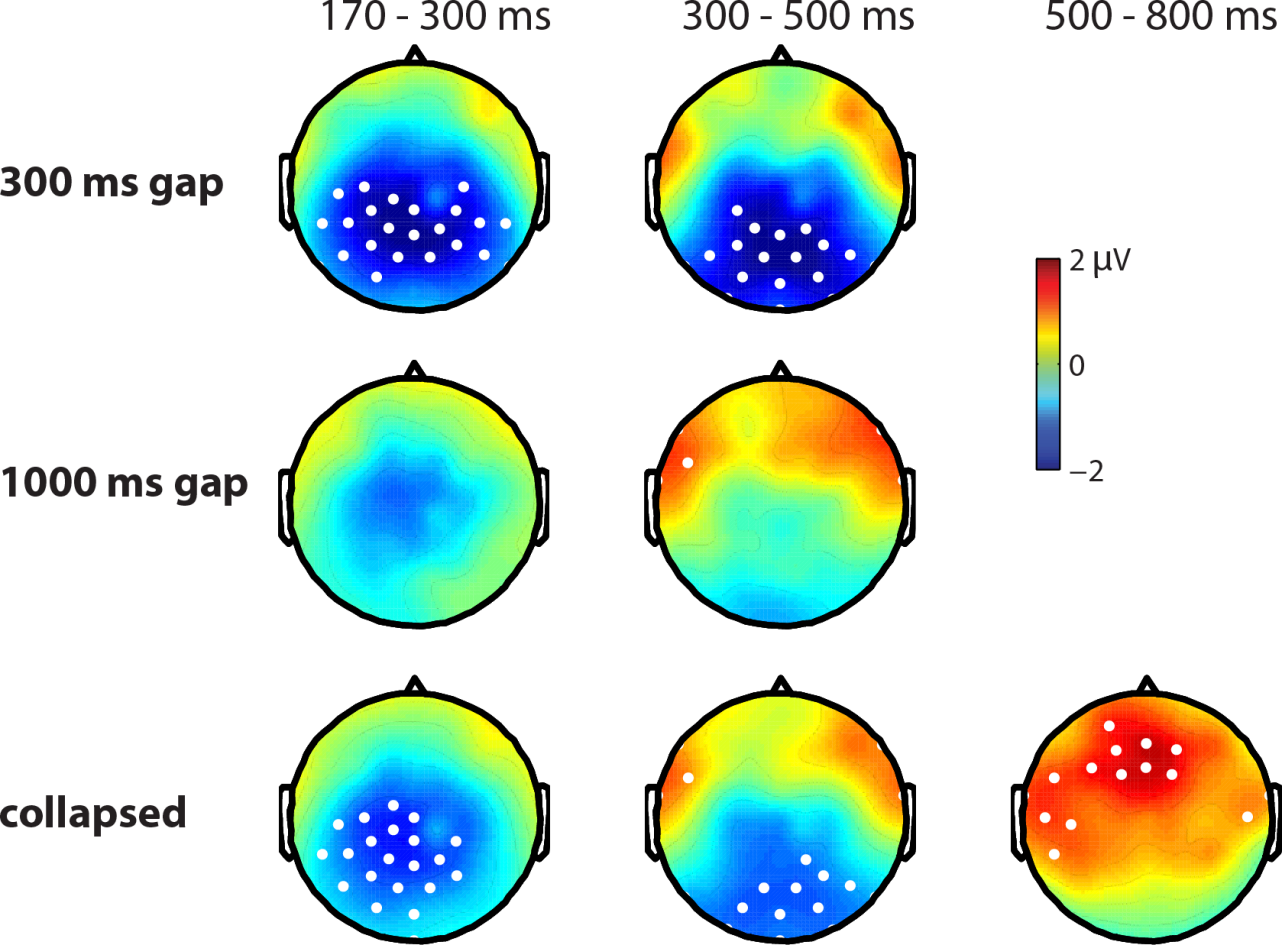
Distribution plots of differences in grand average waveforms between 'no' and 'yes' responses (in microvolts) for three consecutive time windows: an early time window (170–300 ms), the standard N400 time window (300–500 ms) and a time window for the late positivity (500–800 ms). The top row shows differences after a 300 ms gap, the middle row after a 1000 ms gap and the bottom row shows collapsed differences over the two gap durations. Electrodes that are significant in at least 70% of the time window are highlighted in white.

A cluster analysis (see Data Analysis) between 0 and 1000 ms, of the interaction between response type and gap duration, yielded only a marginal interaction between 370 and 486 ms (p = .1). However, a similar analysis in the pre-defined N400 time-window (300–500 ms, see Data Analysis) yielded a significant cluster in the same time range (p = .032). Examining this interaction for the different gap durations, we found a negative cluster for 'no' vs. 'yes' responses after a 300 ms gap in the complete 300–500 ms analysis window (p = .006). To see whether this effect might have started earlier, the same analysis was done in a broader window between 0 and 1000 ms, yielding a negative cluster between 168 and 528 ms (p = .006). Although only one cluster was found, Fig 1 suggests that this effect might consist of an early and a late part, especially looking at anterior electrodes. Fig 3 (top row) shows the distribution of the effect in the standard 300–500 ms window and in the earlier part of the window (170–300 ms). The distribution appears to be centroposterior in the N400 window and more central in the first part. This could suggest two separate effects, an early negative effect, followed by an N400 (see Discussion). The cluster analysis in the long gap condition between 300 and 500 ms after response onset yielded a positive cluster for 'no' vs. 'yes' responses in the complete window (p = .02) with an anterior lateral distribution (see Fig 3, middle row). Given the very different distribution from the standard N400, this effect is likely to be an early onset of the positivity visible in Fig 2 (see below) and not an N400 effect for 'yes' vs. 'no'.

No significant interactions were found between response type and gap duration in a cluster analysis over the complete 0–1000 ms window, nor in a more specific analysis of the 500–800 ms window. The absence of an interaction warranted collapsing the same responses after different gap durations. A cluster analysis between 0 and 1000 ms of 'no' vs.' yes' responses yielded a positive cluster between 242 and 796 ms (p = .002), corresponding to the late positivity. Its distribution was mainly anterior (see Fig 3, bottom row). Additionally, a negative cluster between 146 and 582 ms was found (p = .008) which was driven by the N400 effect for 'no' vs.' yes' that was only present for the 300 ms gap (see above).

In summary, we found an N400 effect (and an earlier negative effect) for 'no' vs.' yes' responses after 300 ms gaps but not after 1000 ms gaps. In addition, a later positivity for 'no' vs. 'yes' responses was found irrespective of gap duration.

## Discussion

In the present study we asked whether initiating actions (requests, offers, proposals, and invitations) would elicit expectations for a preferred response and whether a delay before the response would change these expectations. We hypothesized (H1) that a dispreferred ('no') response after the short gap (300 ms) that is the norm in conversation would elicit a larger N400 than a preferred ('yes') response. This was confirmed. We also hypothesized (H2) that this effect would disappear after a long gap–this was also confirmed. We also thought it possible (H3) that a highly delayed (1000 ms) ‘yes’ would be associated with an N400 greater than ‘no’ in the same condition. This cross-over hypothesis, however, was not confirmed. Instead the data show no difference in N400 amplitude between 'yes' and 'no' after a long delay, which can be described as a 'convergence' of the N400 components.

The results thus show that listeners changed their expectations of a positive or negative response purely based on the duration of silence between two turns. In addition to modulations in N400 amplitude, we also found a larger late positivity, frontally distributed, for 'no' than 'yes' responses, irrespective of the gap duration between question and response. We think this effect most likely reflects extra cognitive processing related to a dispreferred response. We will discuss these two findings in turn.

### N400: Changing expectations

The N400 is argued by some researchers to index specific predictions about upcoming lexical items (e.g., [24]) whereas others argue that it reflects the integration of a word ('yes' or 'no' in this case) into the current context (e.g., [25]). In both views, the current model of the context has to be updated in the light of incoming information that is then integrated with the critical word. In the present study, the contextual model is apparently updated and changed by a long gap between turns and therefore becomes relatively more compatible with a dispreferred response.

As previously noted, we found early negative differences between 'no' and 'yes' responses after a 300 ms gap, at some sites already around 170 ms. In auditory ERP studies, such early negative effects have been found when the first phoneme of an unexpected word did not match the first phoneme of the expected word [51, 52]. This is also the case in the present study since the responses were always either a *ja* ('yes') or a *nee* ('no'), providing early evidence for the violation of an expectation of a *ja* ('yes') when a *nee* ('no') is encountered. These early negativities have been described as either an early onset of the N400 (e.g., [53]) or a separate component, termed the N250 [54] or phonological mismatch negativity (PNM [55]). The slightly more central distribution of the early negativity in the present study appears compatible with it being a separate component. Hagoort and Brown [54] interpret the N250 as a reflection of the lexical selection stage in which form information from the current word and content-based information from the context are integrated, whereas the N400 reflects purely content-based integration. In any case, it appears clear that both components index the degree of match between the context and the input. The interest for this study is that an N250 would indicate not only a semantic preference for a response in line with the initiating action but an expectation of a specific form (*ja*) in a specific discourse context, which looks more definitively predictive.

As indicated above, we did not find the hypothesized cross-over interaction (H3) for the N400 effect; rather the N400s for preferred and dispreferred responses converged. This hypothesis was based on the observation that after short gaps preferred responses are more frequent than dispreferreds but after long gaps dispreferred responses are more frequent than preferreds [10]. One possible explanation for the failure of H3 concerns the distribution of responses likely encountered by participants in their daily lives. In general, preferred responses are more common than dispreferreds, and short gaps are more common than long ones [10, 14]. Thus the expectation that preferred responses should occur after short gaps may be stronger than the expectation that dispreferreds should occur after long ones, leading to a smaller or negligible N400 effect. Alternatively, since preferred responses favor the accomplishment of the activity and index social solidarity and affiliation [12, 13], listeners’ expectations may reflect a general bias towards socially affiliative responses [40] over and above the statistical patterns. In that case, such a general tendency to expect a preferred response might balance the delay-induced expectation of a dispreferred response, leading to a net outcome of no N400 effect.

### Late positivity: never say no

In contrast to the N400 effect, the anterior positive effect coming approximately 600 ms after response onset for ‘no’ versus 'yes' responses was present irrespective of gap duration. A descriptively smaller and less widely distributed positivity after the short gap can probably be explained by attenuation of this positivity from partial overlap with the N400. However, no interaction between the two gap durations was found, so the late positive effects for 'no' relative to 'yes' after the two different gaps can be interpreted as equivalent.

Late positivities or P600 effects have been interpreted in different ways (e.g., [42, 43, 44]). Often these interpretations involve reanalysis or extra processing of complex stimuli (e.g., grammatically incorrect or locally ambiguous sentences). Given that the positivity was found as a main effect between 'yes' versus 'no' responses, we cannot exclude the possibility that it is related to a low-level frequency difference between these two response tokens, since *ja* is more frequent than *nee* in Dutch (in the Dutch spoken corpus from which the stimuli were taken [46], *ja* was at least five times as frequent as *nee*). However, research on frequency effects in ERPs have mainly looked at early effects. One study found a larger P300 for high frequency than low frequency words [56]. Low frequency words have also been found to show positive effects relative to high-frequency words, but these effects occurred earlier than in the present study and were mainly posterior in distribution [57, 58], while the present positivity was mainly anterior.

It is also known that negative utterances involve extra processing, typically with longer reaction times [59]. However, recent work shows this processing difficulty to be largely related to contextual fit [60]. When this is properly controlled, ERP effects (typically negative shifts) disappear or are highly attenuated: “relating incoming words to our real-world knowledge is not necessarily more difficult in negated than in affirmative sentences, as long as negation is used to convey a pragmatically sound message” [61] (p. 1217).

Without then attributing the extra integration effort to frequency effects or the semantic difficulties of negation, in our task the dispreferred character of rejections may raise other problems in the minds of our participants. As mentioned, the preference system biases expectations towards socially affiliative responses, so that there are extra social consequences for rejections [12, 13]. For that reason, rejections are usually accompanied by explanations (e.g., *no*, *I have to be at work then*). The rejections in our stimuli, in contrast, had no such accounts attached because we had to control them and match them in duration to the acceptances and compliances. Rejections will be disaffiliative however packaged, but our stimuli perhaps project stronger social consequences–they are rude. Indeed, late (frontal or widely distributed) positivities have previously been associated with violations of social norms, for example, when participants read descriptions of morally unacceptable behavior [34] or statements that contrast with their moral values [35]. Our result also fits with a recent ERP study showing a frontal positivity for an indirect rejection of an offer (e.g., *do you want me to pay for the ticket?—I have a credit card*) relative to a more neutral answer to a question [38]. These frontal positivities might thus reflect the socially disaffiliative nature of rejection–a *dispreference effect*, as it were. Rejections in conversation are also often preceded by turn-initial particles (e.g., *well* in English or *nou* in Dutch), and the absence of such particles in our stimuli could have resulted in a mismatch of specific lexical predictions, which has been shown to produce late frontal positivities [41].

## Conclusion

This is one of the first studies to show that the N400 can not only index expectations within an utterance or a monologic discourse but can also reflect expectations over the turns of different speakers in a conversation. The results show an effect of the preference structure imposed by initiating actions on the expected responses, by yielding an N400 effect for dispreferred relative to preferred responses after a short gap. This effect disappears after a long gap, indicating that expectations about the response are changed by the sheer duration of the silence before the response is given.

The fact that listeners anticipate a response already before it occurs might constitute one reason for the speed of turn-taking in conversation, where transitions between turns are remarkably short (see, e.g., [14, 62]). Speakers in conversation are faced with the problem of quickly having to interpret the current turn and then prepare an appropriate response, which should then be delivered on time–a problem that can only be solved by prediction [5, 37].

In trying to understand the late positive effect common to the negative responses regardless of timing, we have suggested that extra processing is required to compute the negative social consequences of rejections to invitations, requests, suggestions and offers, especially when the rejection is not accompanied by mitigating particles or explanations. The preference system, ranking response types, is so designed to avoid these negative consequences where possible.

There is still relatively little experimental exploration of interactive language use using EEG. We have used ecologically valid materials, with actual turns extracted from conversations, but our participants were third parties overhearing short dialogues, rather than actual participants in a conversation. The effects found in the present study might be even stronger when the responses are directed to participants themselves in a 'second-person perspective' (see, e.g., [63]), a direction future neuro-imaging research on dialogue could usefully take [37].
